# Understanding COVID-19 Impacts on the Health Workforce: AI-Assisted Open-Source Media Content Analysis

**DOI:** 10.2196/53574

**Published:** 2024-06-13

**Authors:** Anita Pienkowska, Mathieu Ravaut, Maleyka Mammadova, Chin-Siang Ang, Hanyu Wang, Qi Chwen Ong, Iva Bojic, Vicky Mengqi Qin, Dewan Md Sumsuzzman, Onyema Ajuebor, Mathieu Boniol, Juana Paola Bustamante, James Campbell, Giorgio Cometto, Siobhan Fitzpatrick, Catherine Kane, Shafiq Joty, Josip Car

**Affiliations:** 1 Lee Kong Chian School of Medicine Nanyang Technological University Singapore Singapore; 2 College of Computing and Data Science Nanyang Technological University Singapore Singapore; 3 Human Resources for Health Policies and Standards Unit Health Workforce Department World Health Organization Geneva Switzerland; 4 Data, Evidence and Knowledge Unit Health Workforce Department World Health Organization Geneva Switzerland; 5 Health Labour Market Unit Health Workforce Department World Health Organization Geneva Switzerland; 6 Director’s Office Health Workforce Department World Health Organization Geneva Switzerland

**Keywords:** World Health Organization, WHO, public surveillance, natural language processing, NLP, artificial intelligence, AI, COVID-19, SARS-COV-2, COVID-19 pandemic, human-generated analysis, decision-making, strategic policy, health workforce, news article, media content analysis, news coverage, health care worker, mental health, death risk, intervention, efficiency, public health, surveillance, innovation, innovative method

## Abstract

**Background:**

To investigate the impacts of the COVID-19 pandemic on the health workforce, we aimed to develop a framework that synergizes natural language processing (NLP) techniques and human-generated analysis to reduce, organize, classify, and analyze a vast volume of publicly available news articles to complement scientific literature and support strategic policy dialogue, advocacy, and decision-making.

**Objective:**

This study aimed to explore the possibility of systematically scanning intelligence from media that are usually not captured or best gathered through structured academic channels and inform on the impacts of the COVID-19 pandemic on the health workforce, contributing factors to the pervasiveness of the impacts, and policy responses, as depicted in publicly available news articles. Our focus was to investigate the impacts of the COVID-19 pandemic and, concurrently, assess the feasibility of gathering health workforce insights from open sources rapidly.

**Methods:**

We conducted an NLP-assisted media content analysis of open-source news coverage on the COVID-19 pandemic published between January 2020 and June 2022. A data set of 3,299,158 English news articles on the COVID-19 pandemic was extracted from the World Health Organization Epidemic Intelligence through Open Sources (EIOS) system. The data preparation phase included developing rules-based classification, fine-tuning an NLP summarization model, and further data processing. Following relevancy evaluation, a deductive-inductive approach was used for the analysis of the summarizations. This included data extraction, inductive coding, and theme grouping.

**Results:**

After processing and classifying the initial data set comprising 3,299,158 news articles and reports, a data set of 5131 articles with 3,007,693 words was devised. The NLP summarization model allowed for a reduction in the length of each article resulting in 496,209 words that facilitated agile analysis performed by humans. Media content analysis yielded results in 3 sections: areas of COVID-19 impacts and their pervasiveness, contributing factors to COVID-19–related impacts, and responses to the impacts. The results suggest that insufficient remuneration and compensation packages have been key disruptors for the health workforce during the COVID-19 pandemic, leading to industrial actions and mental health burdens. Shortages of personal protective equipment and occupational risks have increased infection and death risks, particularly at the pandemic’s onset. Workload and staff shortages became a growing disruption as the pandemic progressed.

**Conclusions:**

This study demonstrates the capacity of artificial intelligence–assisted media content analysis applied to open-source news articles and reports concerning the health workforce. Adequate remuneration packages and personal protective equipment supplies should be prioritized as preventive measures to reduce the initial impact of future pandemics on the health workforce. Interventions aimed at lessening the emotional toll and workload need to be formulated as a part of reactive measures, enhancing the efficiency and maintainability of health delivery during a pandemic.

## Introduction

The health workforce is a critical component of any health system [1]. Previous studies have suggested a positive correlation between health expenditure and population mortality rates [2]. The projection for 2020-2030 suggests that the health expenditure share for the health workforce will continue to dominate [3]. Policy makers are increasingly interested in the relationship between investments in the health workforce and better health outcomes and the effectiveness of such investments [4,5].

The COVID-19 pandemic has had profound effects on the health workforce worldwide. These impacts include increased morbidity and mortality due to insufficient personal protective equipment (PPE) [6], higher rates of mental health issues such as burnout, stress [7-10], poor sleep quality [11], and negative professional and personal identities [12] to the point of leaving the profession [13]. This unprecedented event reaffirms the need for reflection on approaches and policies toward the health workforce, focusing on comprehensive, reliable, and strategic intelligence to support decision-making efforts [14] to enhance health systems resilience [15].

To better assess and measure the impact of the COVID-19 pandemic on the health workforce, the World Health Organization (WHO) developed a global approach including a framework for standardized measurement and reporting and interim guidance [16]. All the collective information around the COVID-19 pandemic and human resources for health resulted in a resolution on the Global Health and Care Compact (World Health Assembly Resolution WHA75/13 [17]). In addition, living systematic reviews aiming at information gathering and analytics on the health workforce in the context of the COVID-19 pandemic to support policy dialogue and advocacy opportunities have also been undertaken [18-22]. Complementary to the living systematic reviews, the WHO has been collecting strategic intelligence on the health workforce related to the COVID-19 pandemic from open sources in the form of Epidemic Intelligence through Open Sources (EIOS) system [23].

With the vast and constantly growing volume of information available on the internet, open-source intelligence [24], has become a novel component in gathering intelligence from a diverse range of sources, such as mass media, government data, professional publications, and commercial data [25,26]. Analysis of publicly available content constitutes part of open-source intelligence. Media content analysis on the impacts of the COVID-19 pandemic on the health workforce has primarily focused on the experiences of the health workforce (eg, perception of safety [27-29], mental distress [28,30-33], labor shortages [30], and online health services and outreach initiatives [34-37]), a comprehensive overview of the disruption caused by the pandemic is still needed.

Recent advancements in artificial intelligence (AI) and its subfields, such as machine learning (ML)—a computational technique that can learn and improve from experience—and natural language processing (NLP)—a computational technique used for the analysis of natural language and speech, present new opportunities for intelligence gathering and analysis. AI research on the COVID-19 pandemic has focused on early warning, pathogen classification, risk assessment, source identification, hotspot detection, tracking and forecasting treatment monitoring, case, and mortality projections, contact tracing, and drug and vaccine development [38-42]. However, ML methods have yet to be used for health workforce intelligence.

Health Workforce Intelligence from Open Sources (WIOS), presented in this paper, allows us to systematically scan intelligence from media that are usually not captured or gathered through structured academic channels, including peer-reviewed journals, and indexed gray literature databases. This study aimed to investigate various impacts of the COVID-19 pandemic on the health workforce, contributing factors to the pervasiveness of the impacts, and policy responses, as depicted in publicly available news articles. To accomplish this objective, we developed an AI tool and implemented a meticulous analysis protocol. Our dual focus was to investigate the impacts of the COVID-19 pandemic and, concurrently, assess the feasibility of rapidly gathering health workforce insights from open sources.

## Methods

### Search Strategy and Data Preparation

The data for this study were extracted from the EIOS system [23], which collects open-source intelligence from over 13,000 sources, including more than 12,000 news outlets and 744 social media accounts. Overall, EIOS collates news articles, social media posts, reports, and other types of content. The EIOS system is provided under the WHO-led EIOS initiative [23], a partnership between different public health stakeholders around the world. By developing a single all-hazards, One Health strategy for early detection, verification, evaluation, and transmission of public health concerns using publicly available information, it pulls together new and current programs, networks, and systems to increase public health intelligence. The EIOS system has been created as a result of collaboration between WHO and the Joint Research Centre of the European Commission. The data set in this study consisted of publicly available news articles published between January 2020 and June 2022, categorized under “Coronavirus,” and filtered by health workforce group, yielding a total of 3,299,158 news articles in English from 243 countries and territories (list and several country tags were established by EIOS) and more than 3000 internet sources.

**Figure 1:**
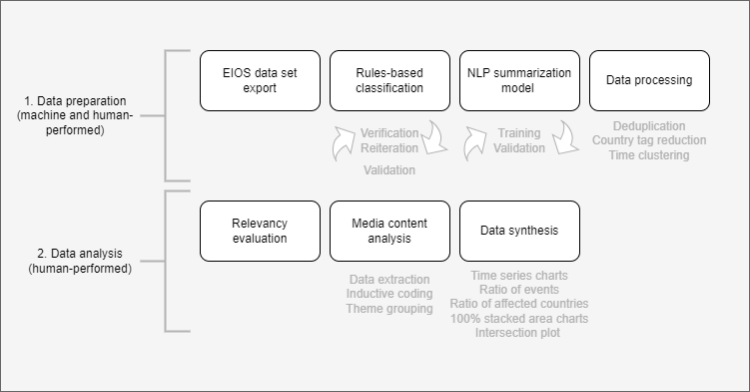
WIOS framework: methods used in data preparation and AI-assisted media content analysis. AI: artificial intelligence; EIOS: Epidemic Intelligence through Open Sources; NLP: natural language processing; WIOS: Health Workforce Intelligence from Open Sources.

To efficiently analyze such a large amount of data, we followed media content analysis [43] and developed a novel AI-assisted media content analysis framework, WIOS. The components of the data preparation are illustrated in Figure 1, the process of developing the tool was reported by Ravaut et al [44]. In the first step, the framework used semiautomated rule-based classification and automated language models. Semiautomated classification rules were developed separately for each topic determined deductively. After multiple iterations, the final set of rules included search strings with Boolean operators nested as word-, phrase-, or value-focused inclusion and exclusion criteria for different sections of each record, such as title, body, and sentence level (Table S1 in Multimedia Appendix 1). To verify the validity of the rules-based classification, we randomly sampled 50 data points flagged as positive for each topic. Human annotators (AP and MM) reviewed whether each of these 50 news articles was indeed relevant and any conflicts or discrepancies in coding were discussed by them, leading to a consensus agreement. The relevance rate fluctuated between 70% and 90% across all topics (Table S2 in Multimedia Appendix 1), confirming the high precision of the rules-based classification system.

Next, we used state-of-the-art NLP models for automated language analysis, specifically, the pretrained off-the-shelf Bidirectional Encoder Representations from Transformers architecture for Extractive summarization (BERTExt) model [45-47]. The model was trained in 2 phases, first, topic-focused (trained with pseudolabels, automatically extracted sentences based on which the system determined inclusion of the record to each topic) and second, with a mixed focus giving the output of both global and topic-focused summarization. We selected the first 3 sentences in the probability ranking as the final predicted summaries of the news articles.

Finally, deduplication was performed, (ie, identification of the article containing 30% repeated trigrams [3 consecutive words] and their removal), country tag reduction from multiple tags to one (the algorithm crosschecked mentions of the country in the title and text using spaCy (Explosion AI) [48] with multiple country tags to identify the most relevant one), and time cluster indication to further facilitate analysis.

Although this report considers news articles over 30 months, the system is designed to enable snapshot analysis on an ad hoc basis and, therefore, provide real-time evidence. This ad hoc functionality requires human supervision, as its core operation incorporates ML components that are routinely augmented by humans. The details of WIOS approach development are available in Ravaut et al [44].

### Data Analysis

Before data analysis, relevancy evaluation has been performed by humans to exclude news articles irrelevant to deductively determined topics. The components of the data analysis are depicted in Figure 1. Deductive-inductive approach was implemented using a proposed qualitative and quantitative media content analysis framework, allowing us to examine the occurrence of selected subtopics [49,50]. The deductive approach produced 5 topics of concern: deaths and infections, mental burden, industrial actions, vaccination, and medical education. Subsequent data extraction and analysis of the content of summarizations, performed in Microsoft Excel, allowed us to generate inductive codes, which were further grouped under 3 themes—areas of impact, contributing factors, and policy responses.

An index of codes was developed based on the key messages extracted from the summarization to capture the essence of each theme. Thereafter, under each area of impact, records have been scanned to identify events contributing to the pervasiveness of impacts. Next, all records about responses were scanned as one group to organize a common index of terms describing responses. Similarly, all records about contributing factors were scanned, overlapping codes merged, and a common index of codes was created.

Finally, the synthesis of data was implemented so that for the areas of COVID-19 impacts, time series charts were developed to show the intensity of media coverage across the topics along with the ratio of the main events, and pie charts were developed for the number of countries affected. For contributing factors and responses, 100% stacked area charts and intersection plots were crafted.

### Ethical Considerations

There were no human participants in this study, and all data were collected from open-source media. Therefore, this study did not require ethics approval.

## Results

### Overview

The preliminary data set exported from EIOS comprising 3,299,158 news articles from January 2020 to June 2022 was reduced through the process of semiautomated data classification to 7674 news articles with 4,629,750 words equivalent to 15,432 standardized pages (see Figure S1 in Multimedia Appendix 1). Data processing and relevance evaluation scaled the data set down to 5131 news articles with 3,007,693 words equivalent to 10,025 standardized pages. Using a summarization model allowed us to reduce the length of each article to 3 sentences resulting in scaling down the data set to 496,209 words equivalent to 1654 pages that facilitated agile analysis performed by humans.

**Figure 2:**
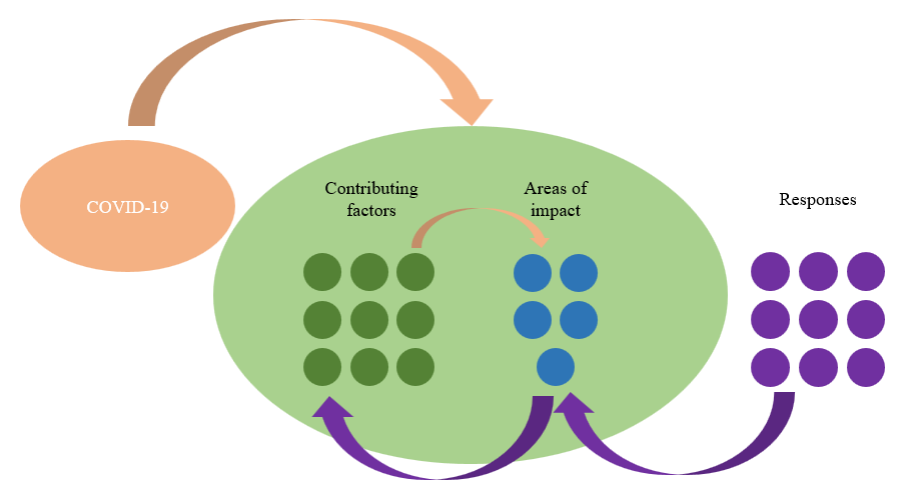
Media content analysis themes for COVID-19 impacts on the health workforce.

The results are shown in 3 sections—areas of COVID-19 impacts and their pervasiveness, contributing factors to COVID-19–related impacts, and responses to the impacts (Figure 2). A visualization of the volume of news articles by month; countries’ subevents according to 5 COVID-19–related areas of impact on the health workforce, including infections and deaths, mental health impacts, industrial actions, and strikes; disruptions of health professionals’ education; and vaccine rollout Figure 2. A further domain analysis comprised factors contributing to COVID-19 impacts and the policy and management actions adopted in response to the identified challenges. Policy responses were often timed, designed, and adopted to respond to multiple impacts and concerns simultaneously.

### Areas of COVID-19 Impacts

**Figure 3:**
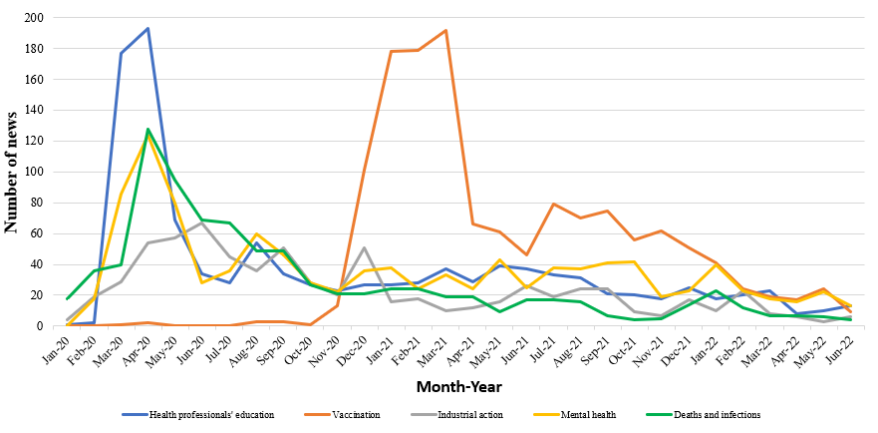
Number of news articles reported monthly on COVID-19 impacts on health professionals’ education, vaccination, industrial action, mental health, and deaths and infections globally (January 2020-June 2022).

Figure 3 shows that the highest volume of news articles on COVID-19 impacts on the health workforce was observed in April 2020 (n=501), and the lowest volume of news articles occurred in June 2022 (n=45). Initially, news articles predominantly focused on health workforce infections and deaths (n=18) in January 2020, but this topic was soon surpassed by health professionals’ education from February to May 2020. Media attention on health professionals’ education gradually decreased and stabilized at fewer than 50 articles per month. Vaccination-related news articles saw a sharp increase since November 2020, reached a peak in March 2021 (n=192), and remained dominant among the 5 topics in the months that followed. The topic of industrial action received relatively the least media attention, with the peak observed in June 2020 (n=69). News articles on mental health, deaths, and infections followed a similar pattern over the 30-month reporting period, with slightly more reports on mental health than on deaths and infections since November 2020. The number of reports on mental health and deaths and infections increased exponentially from January 2020 to a peak in April 2020, after which they declined. Figure S2 in Multimedia Appendix 1 depicts the number of countries with news articles on each topic and follows the pattern shown in Figure 3.

Table 1 displays the distribution of subevents categorized according to the 5 COVID-19 impact topics from January 2020 to June 2022. Among the health professionals’ education subevents, the most frequently reported event was the suspension of clinical training (42/137, 30.7%), followed by the suspension of in-person classes (30/137, 21.9%) and exam postponement (23/137, 16.8%). Half of the news articles on vaccination did not specify vaccination status (78/156, 50.0%), while one-third (49/156, 31.4%) discussed events related to receiving the first dose, and 16.0% (25/156) discussed full or second vaccination. Concerning industrial action, 51.0% (128/251) of the news articles discussed demonstrations, pickets, and protests, while the remaining 42.6% (107/251) and 6.4% (16/251) discussed strikes, sit-downs, or walkouts, respectively. Exhaustion, overwork, and fatigue were the most frequently discussed mental health issues (332/1162, 28.57%), followed by stress (238/1162, 20.48%), burnout (209/1162, 17.99%), and anxiety (166/1162, 14.29%). Nearly 53.9% (307/570) of the articles reported infections, 23.7% (135/570) of the articles reported deaths, and 22.5% (128/570) reported both infections and deaths. Figure S3 in Multimedia Appendix 1 illustrates the number of countries with at least 1 news article mentioning each subevent during the studied period.

**Table 1:** Distribution of subevents of health professionals’ education, vaccination, industrial action, mental health, and deaths and infections impacted by COVID-19 globally (January 2020-June 2022).

Subevents for each area of COVID-19 impacts		Frequency of reporting, n (%)
**Infection and death (n=570)**		
	Infections	307 (53.9)
	Deaths	135 (23.7)
	Infections and deaths	128 (22.5)
**Health professionals’ education (n=137)**		
	Exam postponement	23 (16.8)
	Suspension of clinical training	42 (30.7)
	Suspension of in-person classes	30 (21.9)
	Suspension of in-person celebrations	5 (3.6)
	Visa issues (study and work)	19 (13.9)
	Other disruptions	18 (13.1)
**Vaccine (n=156)**		
	First dose	49 (31.4)
	Second dose or fully vaccinated	25 (16.0)
	Booster dose	4 (2.6)
	Unspecified	78 (50.0)
**Industrial action (n=251)**		
	Demonstration, picket, or protest	128 (51.0)
	Sit-down or walkout	16 (6.4)
	Strike	107 (42.6)
**Mental health (n=1162)**		
	Anxiety	166 (14.3)
	Burnout	209 (18.0)
	Depression	65 (5.6)
	Exhaustion, overwork, and fatigue	332 (28.6)
	Sleep issues	36 (3.1)
	Stress	238 (20.5)
	Suicide	24 (2.1)
	Trauma, PTSD^a^, and STD^b^	92 (7.9)

^a^PTSD: post-traumatic stress disorder.

^b^STD: secondary traumatic stress.

Table 2 presents the number of affected health workforce by year and selected topics. Among news articles that reported numbers of affected individuals, the number of health workforce who received COVID-19 vaccinations surged from 19,002 in 2020 to a staggering 62,767,012 in 2021. According to the media reports, slightly over 2 million health workforces received a COVID-19 vaccination in the first half of 2022. The number of health workforce on strike fell from more than 3.6 million in 2020 news articles (n=3,610,735) to around 100,000 in 2021 and 43,000 in the first half of 2022. The media reported 2,574,467 infections among the health workforce in 2020, which decreased to nearly half that number in 2021 and was 25 times lower in the first half of 2022. Similar to infections, media-reported deaths among the health workforce exceeded 31,000 in 2020, decreasing to slightly more than 25,000 in 2021. This dropped further to 653 in the first half of 2022. The number of health workers affected by mental health burdens and disruptions in health professionals’ education could not be ascertained due to a lack of data in included media articles.

**Table 2:** Number of health workers affected by COVID-19 regarding vaccination, strikes, infections, and deaths (January 2020-June 2022).

Areas of COVID-19 impacts	2020, n	2021, n	2022, n
Vaccine	19,002	62,767,012	2,046,596
Strike^a^	3,610,735	108,211	43,000
Infections	2,574,467	1,297,837	50,840
Death	31,111	25,364	653

^a^The majority of the health workforce involved in strikes in 2020 was in India (n=3,500,000).

### Factors Contributing to COVID-19 Impacts

**Figure 4:**
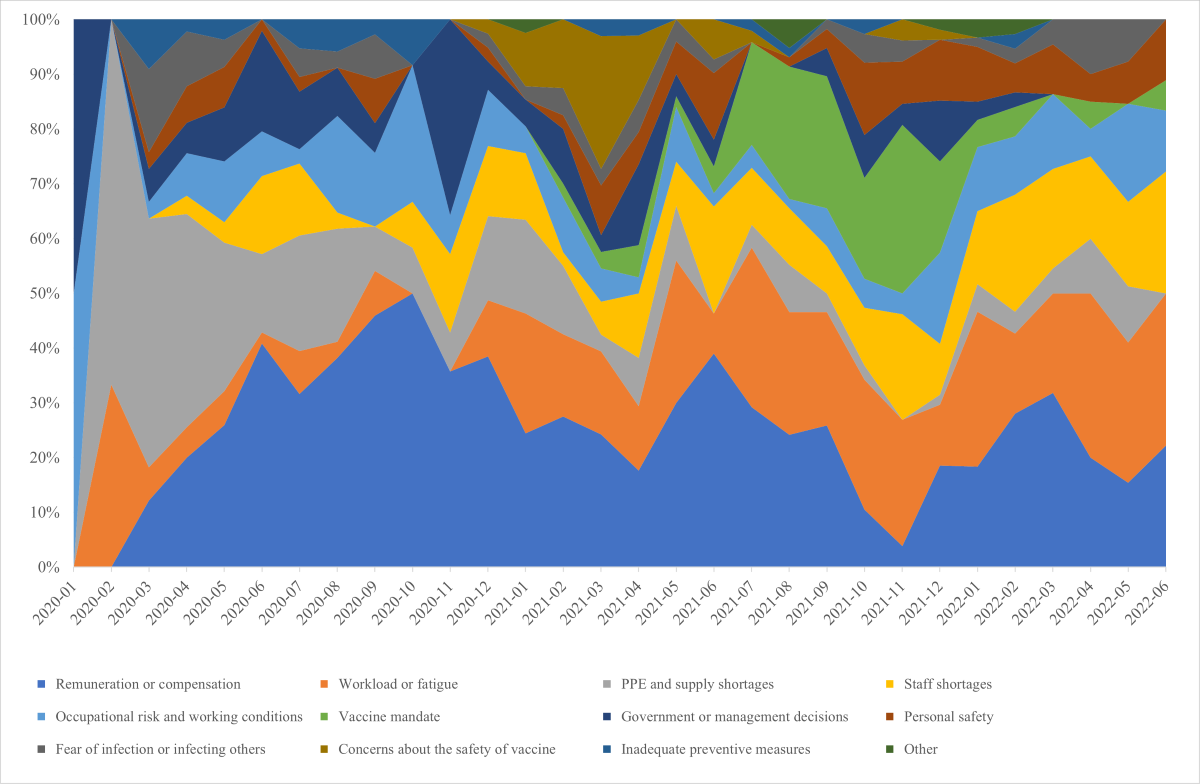
Contributing factors to COVID-19–related impacts on the health workforce (January 2020-June 2022). PPE: personal protective equipment.

**Figure 5:**
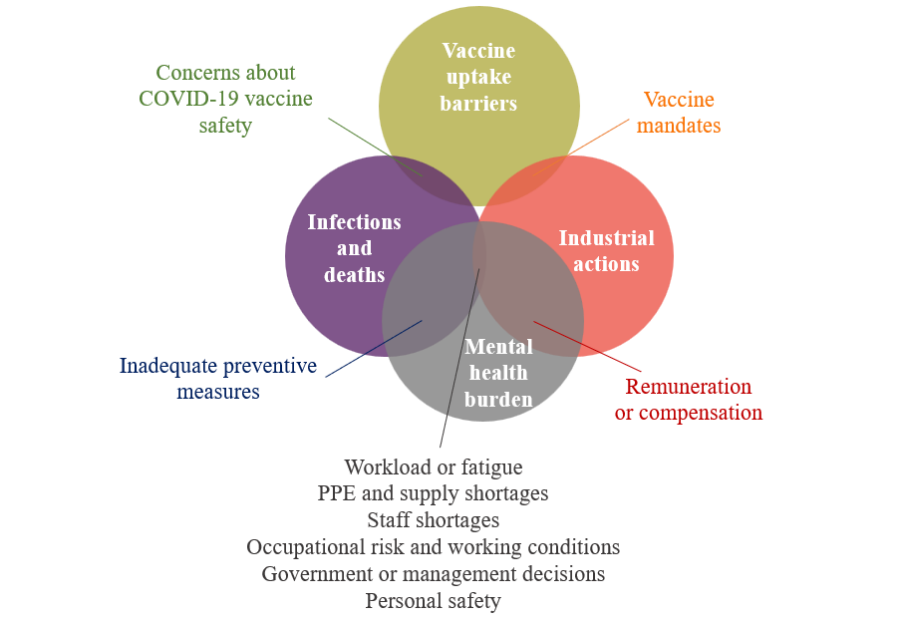
The overlap of reported contributing factors to COVID-19–related impacts on the health workforce (January 2020-June 2022). PPE: personal protective equipment.

While specific figures may be available through government or employer databases and reporting, our analysis identified 12 categories related to the factors contributing to COVID-19 impacts on the health workforce, visualized on a 100% stacked area chart according to months (Figure 4) and their overlap with respective topics (Figure 5).

Challenges related to remuneration and compensation, including inadequate wages, delayed payments, lack of overtime pay, hazard pay, and insurance, were the most commonly reported factors contributing to both industrial action and mental health impacts in the news sources assessed. Articles on these challenges first peaked in March 2020 and remained high with the highest peak in October 2020.

Excessive workload, lack of adequate PPE, staff shortages, occupational risks, government policies, and personal safety were important concerns reported by news articles on the health workforce’s industrial actions, infections, deaths, and mental health burdens. Reports about lack of or inadequate PPE dominated in the first quarter of the pandemic but were reported less in the later stages of the COVID-19 pandemic, while concerns regarding occupational risks persisted throughout the whole study period. Policy and management decisions such as fund misappropriation, budget cuts, conflict with hospital management, dissatisfaction with the relaxation of public health measures, and underappreciation were reported as factors contributing to the impacts during the first year of the pandemic, with a spike observed in November 2020. Staff shortages were widely reported throughout the pandemic but gained more attention in public discourse in the second year. Similarly, we noticed an increase in the influence of workload and fatigue in news articles beginning in mid-2021. Furthermore, since the beginning of the second year of the pandemic, there has been an increase in the impact on personal safety, including incidences of harassment, victimization, and stigmatization, with spikes in June and October 2021, as well as January and June 2022.

Media reports of concerns about vaccine safety, such as skepticism surrounding the quality of different vaccine brands or fear of side effects may have had an impact on infections and deaths of the health workforce, as well as vaccine hesitancy, from late 2020 to mid-2021, with a peak in March 2021. The concerns faded while dissatisfaction with vaccine mandates surfaced, contributing to industrial actions and vaccine hesitancy from July 2021 to December 2021. Inadequate preventive measures, such as lack of safety protocols or COVID-19 testing, were identified as potential contributing factors for infections or deaths throughout the first year, while the fear of getting infected or infecting others impacted mental health burdens persistently yet subtly over time.

### Responses to COVID-19 Impacts

**Figure 6:**
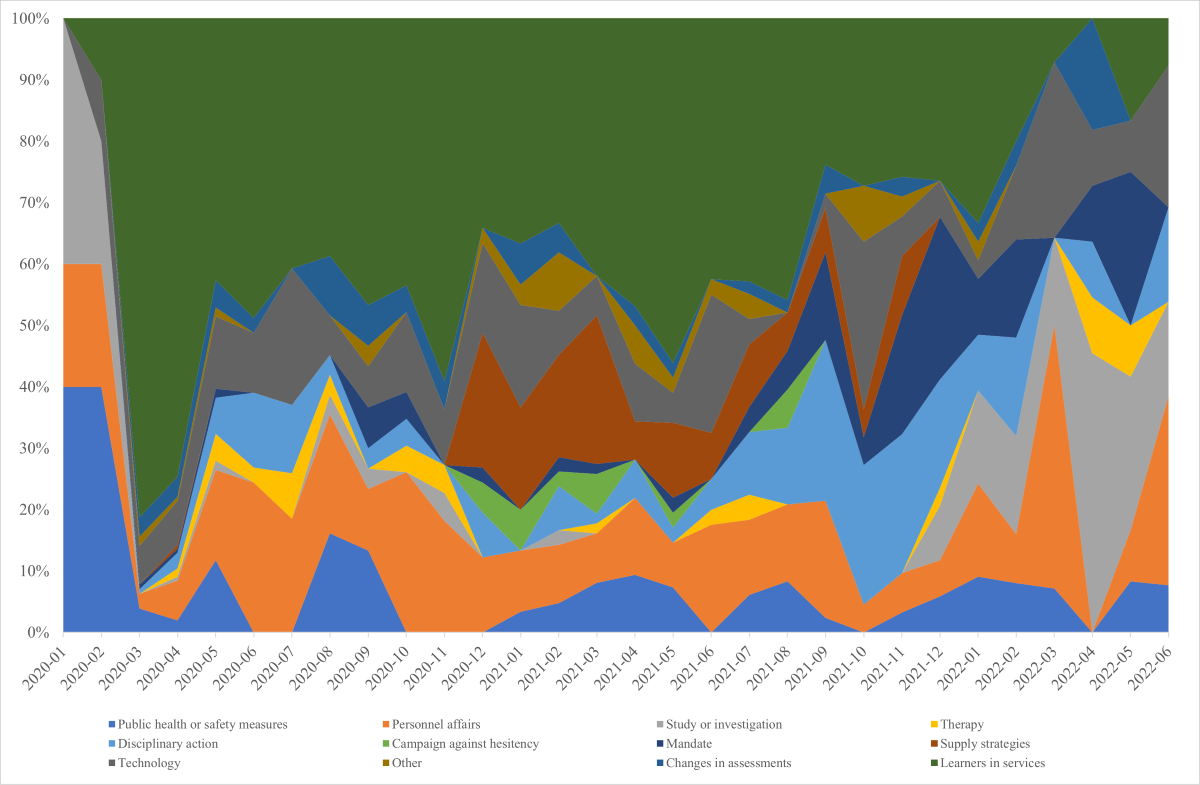
Responses to COVID-19–related impacts on the health workforce (January 2020-June 2022).

**Figure 7:**
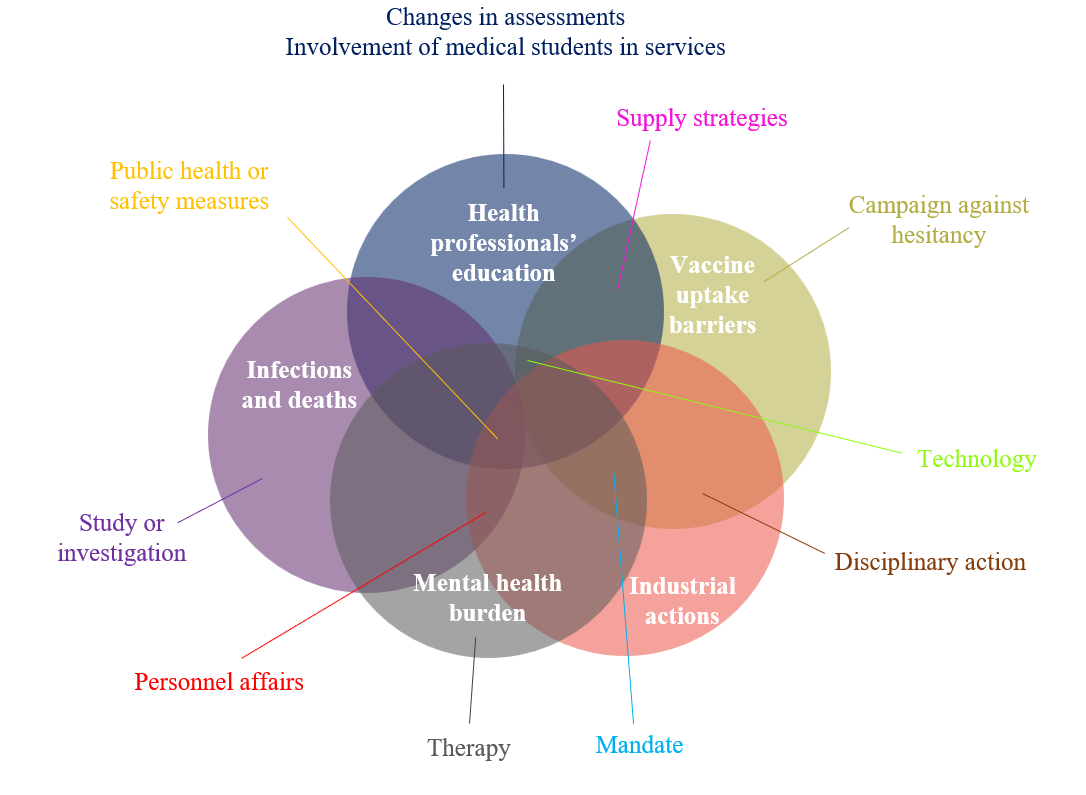
Overlap of responses to COVID-19–related impacts on the health workforce (January 2020-June 2022).

Figure 6 is a 100% stacked area chart that displays 12 health workforce policy and management responses to the COVID-19–related impacts on the health workforce, organized by month, while Figure 7 presents their overlap with respective topics.

Our analysis revealed that policy and management responses included students supporting service provision teams, managing personnel affairs (including salary increments, insurance, hazard pay packages, etc), technology, disciplinary action, implementing public health or safety measures, supply strategies, and enforcing mandates had an impact on 4 out of 5 themes—medical education, infections and deaths, vaccine uptake, and industrial action. In the first year of the pandemic, the primary policy and management responses were medical students supporting service provision teams, personnel affairs, technology, and public health or safety measures. However, their impact decreased in subsequent years.

Narratives of disciplinary action, such as sanctions and arrests, surfaced as a means of mitigating challenges during the pandemic. This response first emerged in March 2020, with articles and reports more than doubling in 2021 and peaking in September 2021. Discussions about mandates, including vaccination mandate or strike prohibition, began in March 2020 and demonstrated varied patterns throughout the year. A subtle yet discernible increase in news reports was observed from October 2021. In December 2020, there was a significant upswing in the influence of various supply strategies reported by news outlets, including emergency approval, small weekly shipments, direct contact with suppliers, priority group allocation, and others. This trend continued in 2021, with the most considerable spike in media reports occurring in March of that year. Throughout the year 2020, news reports referred to therapeutic approaches like counseling, psychotherapy, and psychosocial support to alleviate or address mental health impacts on the health workforce. The peak in reporting was seen in May 2020, followed by a 2-year slow drop. Adjustments in assessments were acknowledged as necessary to continue health professionals’ education during the first year of the pandemic, but their importance faded in the following years. Study and investigation evolved as responses to problems encountered throughout the pandemic. The response appeared at the onset of the pandemic and exhibited fluctuating patterns of news reporting in 2020, with a sudden drop in 2021 but continued for 6 months in 2022. Another critical response identified was the campaign to improve vaccine uptake and promotion, which was initiated in December 2020, peaked in March 2021, and disappeared after August 2021.

## Discussion

### Primary Findings

To examine how the media depicted the diverse impacts of the COVID-19 pandemic on the health workforce, we conducted a comprehensive analysis of over 3 million news articles from January 2020 to June 2022, collected by the EIOS platform. We applied NLP techniques to synthesize relevant information from a large volume of news articles. We focused on 5 key domains that captured the effects of the COVID-19 pandemic on the health workforce—infections and deaths, vaccination, industrial action, mental health, and health professionals’ education. We also explored the factors that influenced these effects and the policy responses that were taken to address them. We identified and analyzed the subevents that occurred within each domain and traced their temporal evolution to the COVID-19 outbreaks. This work follows the shift toward new approaches to strategic health workforce intelligence [51] by using open-source data, the NLP approach, and human analysis.

The health workforce who were at the forefront of the response to the COVID-19 pandemic were exposed to a grave danger that threatened their health and survival. Our media content analysis documented the trends and patterns of infections and deaths among this population, showing a gradual decrease over time. However, it also identified the factors that contributed to these adverse outcomes, such as suboptimal preventive measures, inadequate testing, and inherent occupational hazards. The analysis also highlighted the wider implications of infections and deaths among the health workforce, which had repercussions not only for their families, coworkers, and communities but also for the health system and the overall pandemic response.

The COVID-19 pandemic laid bare and exacerbated pre-existing challenges faced by the health workforce, encompassing issues such as inadequate compensation, delayed payments, absence of overtime pay, hazard pay, and insurance, excessive workloads, insufficient PPE, staff shortages, occupational risks, and personal safety concerns. Our media content analysis documented the diverse forms of collective action undertaken by the health workforce to advocate for improved working conditions, recognition, and protection. Mainly, demonstrations, protests, and strikes emerged as common manifestations of such actions, unfolding across disparate countries and regions throughout the pandemic. The media analysis also highlighted instances where the health workforce faced disciplinary measures, sanctions, or arrests for participating in or organizing industrial action.

The development and dissemination of COVID-19 vaccines represented a watershed moment in the global response to the pandemic. Our media content analysis discerned a steady surge in the vaccination of the health workforce over time. Despite this progress, challenges persisted, manifesting in concerns related to vaccine safety, side effects, and mandates. Notably, hesitancy or reluctance among the health workforce, coupled with barriers to access and discrimination in vaccine distribution, emerged as notable themes. Additionally, varying levels of acceptance and availability of different vaccine brands were observed, contingent upon geographical location.

The toll of the COVID-19 pandemic on the mental health and well-being of the health workforce was also profound, as they faced unprecedented levels of stress, pressure, and uncertainty in their occupational roles. Our media content analysis revealed common mental health issues were reported in news articles, including exhaustion, overwork, fatigue, stress, burnout, anxiety, and fear of infection. These issues had repercussions on the performance, motivation, and morale of the health workforce, harming both personal and professional relationships. Although therapeutic interventions such as counseling, psychotherapy, and psychosocial support were suggested to cope with these difficulties, the analysis highlighted the insufficient availability and accessibility of such services for all health workforce in need.

The unprecedented COVID-19 crisis posed significant challenges to health professionals’ education institutions and programs, affecting both students and educators in various ways. Our media content analysis revealed that the most common events reported in relation to health professionals’ education were the suspension of clinical training, face-to-face classes, exams, and graduation ceremonies. These events had implications for the quality, continuity, and accreditation of medical education, as well as the career opportunities and well-being of students and educators. In response to these difficulties, adaptive measures, such as online learning, remote supervision, alternative assessments, and early graduation, were adopted to cope with the changing situation.

### Comparison With Prior Work

Our findings revealed that media coverage of COVID-19’s impact on the health workforce peaked in April 2020, subsequently waning until June 2022. Following the WHO’s declaration of a Public Health Emergency of International Concern in January 2020 [52], a surge in news articles emerged predominantly addressing health and care worker infections and fatalities. Postdeclaration, all topics except vaccinations gained heightened media attention, with disruptions in health professionals’ education taking center stage for several consecutive months due to suspended clinical training and in-person classes. Additionally, media coverage of health and care worker infections, deaths, mental health strains, and educational disruptions reached a zenith in April 2020. A subsequent surge in vaccination-related news articles occurred in November 2020, overshadowing other topics for an extended period. The subsequent year’s exponential increase in the vaccinated health workforce can be traced back to the announcement of successful COVID-19 vaccine development in the same month [53]. Notably, over 60 million health workforce were reported to have been vaccinated in 2021, resulting in a significant reduction in infections and deaths compared with 2020. Media coverage also documented various forms of activism, such as demonstrations, protests, strikes, and walkouts, peaking in June 2020. Exhaustion and fatigue, often caused by overwork, emerged as the most frequently discussed mental health issues in the health workforce.

Given the global concern surrounding the COVID-19 pandemic’s impact on the health workforce, this study also explored contributing factors and offers future recommendations. Insufficient remuneration and compensation packages, including risk allowances, have been key disruptors for the health workforce during the pandemic, leading to industrial actions and mental health strains. Prior research has identified these factors as significant contributors to pandemic-related disruptions among the health workforce [54]. Additionally, shortages of PPE and occupational risks have profoundly affected the health workforce, increasing infection and death risks, particularly at the pandemic’s onset. Workload fatigue and staff shortages, while less time-sensitive, have intensified as the pandemic progressed. Public discourse on workload and fatigue became increasingly prominent in the pandemic’s second year, with staff shortages contributing to service delivery disruptions. Previous research has linked fear of infection, PPE scarcity, close contact with patients with COVID-19 and heavy workloads to adverse mental health outcomes in the health workforce [55]. Consequently, the findings of this study point to the need to prioritize implementing suitable remuneration and compensation packages, as well as ensuring adequate PPE supplies as preventive measures to lessen the initial impact of future pandemics on the health workforce. Moreover, in the progressing trajectory of pandemics, the health workforce is grappling with escalated duty hours and augmented caseloads. While an investigation into their encounters with heightened intensity and the expanded workload becomes imperative, interventions targeting the emotional toll induced by the demands of caregiving duties need to be formulated, enhancing the efficiency and maintainability of health delivery. Effective planning, training, and resource allocation can facilitate these goals.

As the COVID-19 pandemic has posed numerous global challenges, various responses have been discussed in the news articles as well. To address mental challenges in the areas of impact such as health impacts, industrial actions, and infections, approaches to personnel affairs, including remuneration, compensation, redeployment, and training, have been implemented. Notably, financial compensation and reactive therapeutic support responses were observed with a slight delay from the pandemic’s onset. This delay accentuates the urgency for expedited measures in this domain, aimed at enhanced readiness for prospective pandemics. Timely implementation of financial incentives, the provision of mental health care services, mitigation of shortages, and amplification of psychological support have emerged as pivotal factors in upholding the motivation of health and care personnel, while concurrently alleviating the mental health encumbrances [56]. In addition to financial incentives, disciplinary action has been used to address challenges posed by industrial action and barriers to vaccine uptake, particularly during the pandemic’s second year. Technology has also played a pivotal role in responding to health professionals’ education [18], mental health burdens, and vaccination hesitancy, especially during the pandemic’s first and second years. Our findings revealed that involving medical students in service exhibited the most common response in mitigating pandemic-related challenges. To address health and care worker shortages, medical school graduates were expedited into health services, enabling them to swiftly combat the virus [57]. Overall, the findings emphasize the importance of efficient personnel management, including financial incentives, therapeutic support, work distribution, appropriate staffing levels, and technology, in alleviating the adverse consequences of pandemics.

WHO involved independent teams to work on analyzing intelligence on COVID-19 impacts on the health workforce. One arm of the work involved media content analysis with the use of AI, and the other systematic reviews of academic literature. Our research findings are in line with the current peer-reviewed literature addressing vaccination acceptance and hesitancy among the health workforce [21]. The literature underscores the concerns of these professionals related to vaccine safety, side effects, and mandates. Our approach, complementing the aforementioned study [21], introduced a temporal component that was absent in the initial research, thereby adding value to the overall understanding of the pandemic trajectory and shift of the focus.

The results of our study yielded similar mental health impacts as identified by Fronteira et al [58] including anxiety, depression, burnout, fatigue, and workplace violence. However, media content did not allow the identification of absenteeism or sleep disorders, but allowed for observing changes in the mental health impacts over time, including workload fatigue intensifying as the pandemic progressed. Our study corroborated the findings of a systematic review [18] that the disruption to clinical training was a major challenge for health workers during the pandemic. Both sources highlighted the need for adaptation, citing various policy responses and emergency measures such as online learning, alternative assessments, early graduation, and volunteerism implemented to cope with the crisis. Consistent with findings from a recent systematic review [19] on the impact of industrial actions on the health workforce, inappropriate financial compensation and suboptimal working conditions (eg, lack of medical protection) were some of the key drivers of undertaking industrial actions. Previous literature review further highlighted the importance of strengthening leadership and management capacities at different levels of health systems to address labor relations and dispute resolutions, media content analysis reports incentives, and disciplinary action as means adopted to address such challenges. Our results are consistent with the findings from a systematic review of policy and management interventions [59], particularly interventions that include public health or safety measures, mental health and well-being support through free counseling services, and financial incentives or salary adjustments. Our findings complement the literature by providing an additional perspective on the temporal relationship between policy responses and the COVID-19 pandemic. It is important to note that signals’ detection based on open-source intelligence should not be used as a sole input source for decision-making, strategy, and action, but rather an invaluable complementary activity that both highlights emerging issues and practices and that can be a pathfinder for more rigorous research and investigation, using standard scientific methodology and practice. Other methodologies, such as systematic reviews and implementation research through mixed methods case studies, are required to assess the results and effectiveness of investment, policy, and management decisions adopted by countries, employers, professional associations, and other relevant stakeholders signaled by open-source intelligence.

### Strengths and Limitations

Our results highlight the news article’s capacity of open-source (publicly available) media as a substantial information reservoir for investigating the effects of adverse public health events on health personnel, insights that might not be procurable elsewhere. The envisaged AI-facilitated analysis holds the potential to provide an agile, timely, and global view of the impact of the COVID-19 pandemic on the health workforce. AI has been going through fast and impressive developments in the last few years, driven by large language models (LLMs), of which the most auspicious event was ChatGPT’s (OpenAI) release in November 2022. In this work, the underlying AI models powering the information selection rely on fine-tuned language models. These middle-sized models achieve high performance but at the cost of fine-tuning on at least several thousand examples. Recent LLMs such as ChatGPT or GPT-4 [60] hold the promise of performing several tasks including classification and summarization in zero-shot, bypassing the need for any annotation or fine-tuning. However, these LLMs are not open-source and introduce new challenges such as the heavy cost of querying the OpenAI at a large scale, reproducibility, and data governance issues. We leave the exploration of using open-source LLM alternatives such as Llama-2 (Meta AI) [61] to further research work.

However, this study could face selection bias owing to external factors such as the search conducted solely in English, or the social and political environment in a particular country that may impact the estimation of affected individuals and, hence, conclusions. It is important to note that the analysis of publicly available news articles should not be relied upon as a sole source for decision-making; rather, it should be used as a complementary activity to gain an understanding of emerging issues and practices, providing a pathfinder for more rigorous research and investigation. Greater input and engagement with infodemic management could complement information gathering and create a symbiotic environment. Compared with standard reviews of the peer-reviewed and gray literature, the workforce intelligence from publicly available news articles was able to screen a much larger and more diverse range of information sources, that is not encompassed in the typical indexed peer-reviewed literature. The framework also carries the potential of rapid use in the case of subsequent health emergencies to satisfy health workforce intelligence needs. The findings were broadly similar in terms of domains and issues documented, as were the policy responses identified [18-22]. The news and media sources led to an earlier identification of issues and challenges before the formal peer-reviewed literature could document them. In this respect, the adoption of this approach may yield benefits, particularly in terms of more timely identification of issues, thereby serving an early warning function. Conversely, the type of information sources identified did not typically include sufficiently granular data on policy detail, nor on the effectiveness and feasibility of different policy measures adopted, thereby limiting the scope for use for policy and normative purposes.

Additional insights could be gained by adding data on media penetration and uptake, which could be measured in particular through social platforms. Similarly, an additional assessment of article syndication and the overall media market within countries (to determine the proportion of reporting on health workers on the whole) would be useful, as would expanding sources to media used more in low-resource environments, including social media.

Finally, the WHO is at the forefront of efforts to address public health infodemics and disinformation [62], and although the source database (EIOS) comprises more than just media articles that have been used for this study (WIOS), we have applied a verification process performed by humans to scrutinize credibility and relevance. Our methodology also carefully ensures that the precision of news articles analyzed is high following rules crafted by domain experts, and validated by human annotators. These steps helped to reduce to the extent possible, any disinformation linked to unscrupulous sources of information and heavily reduced the risk of misinformation, even if it did not suppress it entirely.

### Conclusions

In summary, the study has highlighted an array of topics relating to the impacts of the COVID-19 pandemic on the health workforce, such as distribution, contributing factors, policy makers’ responses, mental health burdens, industrial actions, vaccinations, and disruptions in medical education. Additionally, the study has shown that AI can support strategic intelligence and decision-making processes through its ability to promptly gather and process information not typically captured by mainstream research approaches or methodologies, for example, systematic reviews.

While the study covers data from January 2020 until June 2022, the overreaching aim of the project was to develop procedures and methods for timely open-source reports and produce regular updates. This study shows the feasibility of applying NLP to classify information for further human-generated analysis and synthesis, which brings about an additional advantage of reducing the lag time of individual observational or interventional studies in the case of future pandemics. Further studies using AI may also extend the search to include sources from other languages. In light of future potential pandemics, it is essential to develop contingency plans and create tools that enable real-time intelligence gathering with multidimensional signal detection for health workforce decision-making: WHO will continue to explore this within the global discussions on the health workforce needs for emergency and pandemic preparedness.

The capacity to track the pandemic impacts on the health and care workforce, the policy responses put in place by governments, employers, and decision-makers, and their results and effectiveness are critical to steering policy dialogue, as well as management and investment decisions. To achieve this, a triangulation of different data sources and evidence methods is necessary, particularly for domains where traditional data sources are known to display gaps in terms of completeness, validity, and timelines. In this context, WIOS represents a promising tool to complement other sources of strategic intelligence.

## Appendix

Multimedia Appendix 1 Search strategy for rules-based classification and detailed results.

## Abbreviations

AI: artificial intelligence
BERTExt: Bidirectional Encoder Representations from Transformers architecture for Extractive summarization
EIOS: Epidemic Intelligence through Open Sources
LLM: large language model
ML: machine learning
NLP: natural language processing
PPE: personal protective equipment
WHA: World Health Assembly
WHO: World Health Organization
WIOS: Health Workforce Intelligence from Open Sources

## Data Availability

The data sets generated and analyzed during this study are available from the corresponding author on reasonable request, subject to World Health Organization approval.
