# Supplementary material for: Understanding COVID-19 Impacts on the Health Workforce: AI-Assisted Open-Source Media Content Analysis
Source: JMIR Form Res. 2024 Jun 13;8:e53574. doi: 10.2196/53574 (PMC11211705; doi:10.2196/53574)
Supplement: Multimedia Appendix 1 [file formative_v8i1e53574_app1.docx]

### Table S1. Search strategy: rules-based classification.

| **Topic: Disruptions, adaptations, and acceleration of health professionals’ education** |
| --- |
| 1. Exclusion |
| 1.1.Remove articles with sentences longer than 100 words (Articles with sentences longer than 100 are noise, e.g., news feeds) |
| 1.2. “NEWS FEED” in the body (We noticed that for this particular topic, there were many articles that start with “NEWS FEED” in the body) |
| 2. Inclusion |
| 2.1. Apply sentence-level inclusive contextual filtering on the body (1/ ensuring that the articles mention education actors or entities 2/ ensuring that the article mentions topics of interest, based on a/ manual recognition of thematic patterns derived from a noisy dataset, b/ multiple iterations of filters): |
| “medical student” or “medical graduate” or “medical studies” or “medical school” or “medical program” or “medical collage” or “medical trainee” or “medical undergraduate” or “medical degree” or “foreign medical graduate” or “foreign medical student” or “international medical graduate” or “international medical student” or “nursing student” or “nursing studies” or “nursing school” or “nursing program” or “nursing college” or “nursing trainee” or “nursing degree” or “dental student” or “dental studies” or “dental program” or “pharmaceutical student” or “pharmaceutical studies” or “pharmaceutical program” |
| AND |
| “graduate early” or “graduating early” or “early graduation” or “early exit” or “early registration” or “fast-track” or “fast track” or “final year” “e-learning” or “online learning” or “recruit” or “suspend” or “cancel” or “exam “ or “exam.” or “exam,” or “online” or “seats” or “volunte" or “visas” or “ visa ” or “not permitted” or “permit or “license” or “admission” or “capacity” or “accelerat” or “placement” |
|  |
| **Topic: Barriers, enablers, and coverage of COVID-19 vaccination for health workforce** |
| 1. Exclusion |
| 1.1. Remove articles with sentences longer than 100 words (Articles with sentences longer than 100 are noise, e.g., news feeds) |
| 1. Inclusion |
| 2.1.Apply sentence-level inclusive contextual filtering on the title (Ensuring that the articles focus on two main topics of interest):  “vaccin” or “booster” or “ jab” or “ shot ” or “Pfizer” or "BioNTech" or “Moderna” or “Sinovac” or “Janssen” or “Johnson & Johnson” or “AstraZeneca” or “Covishield” or “Covaxin” or “Sinovac” or “Sinopharm” or “Sputnik” |
| AND professions = [     "health specialist", "health provider", "health professional", "health practitioner", "health doctor", "health worker", "health personnel", "health staff",     "healthcare specialist", "healthcare provider", "healthcare professional", "healthcare practitioner", "healthcare doctor", "healthcare worker", "healthcare personnel", "healthcare staff",     "health care specialist", "health care provider", "health care professional", "health care practitioner", "health care doctor", "health care worker", "health care personnel", "health care staff",     "medical specialist", "medical provider", "medical professional", "medical practitioner", "medical doctor", "medical worker", "medical personnel", "medical staff",     "physician", "general practitioner", "therapist", "nurse", "midwife", "caregiver", "care provider",     "gastroenterologist", "surgeon", "dentist", "psychiatrist", "pharmacist", "ophthalmologist", "cardiologist", "anaesthesiologist",     "anesthesiologist", "dermatologist", "endocrinologist", "geriatrician", "haematologist", "hematologist", "nephrologist",     "neurologist", "oncologist", "otolaryngologist", "pulmonologist", "paediatrician", "pediatrician", "radiologist", "urologist" ] |
| 2.2. Apply sentence-level inclusive contextual filtering on the body (Ensuring that 1/ the articles inform about the coverage of vaccination for HCW or 2/ or mention known barriers perceived by HCW): (“vaccin” or “immuniz” or “immunis” or “booster” or “ jab” or “ shot ” or “Pfizer” or "BioNTech" or “Moderna” or “Sinovac” or “Janssen” or “Johnson & Johnson” or “AstraZeneca” or “Covishield” or “Covaxin” or “Sinovac” or “Sinopharm” or “Sputnik” |
| AND professions = [     "health specialist", "health provider", "health professional", "health practitioner", "health doctor", "health worker", "health personnel", "health staff",     "healthcare specialist", "healthcare provider", "healthcare professional", "healthcare practitioner", "healthcare doctor", "healthcare worker", "healthcare personnel", "healthcare staff",     "health care specialist", "health care provider", "health care professional", "health care practitioner", "health care doctor", "health care worker", "health care personnel", "health care staff",     "medical specialist", "medical provider", "medical professional", "medical practitioner", "medical doctor", "medical worker", "medical personnel", "medical staff",     "physician", "general practitioner", "therapist", "nurse", "midwife", "caregiver", "care provider",     "gastroenterologist", "surgeon", "dentist", "psychiatrist", "pharmacist", "ophthalmologist", "cardiologist", "anaesthesiologist",     "anesthesiologist", "dermatologist", "endocrinologist", "geriatrician", "haematologist", "hematologist", "nephrologist",     "neurologist", "oncologist", "otolaryngologist", "pulmonologist", "paediatrician", "pediatrician", "radiologist", "urologist" ] |
| AND one number (but ignore if “-number”, “number-“ [like in “Covid-19”, “2019-nCoV”, “SARS-CoV-2”], “number+space+min”, “number+space+sec”, “number/”, “number year old”, “number years old”, “number,+space”, “number:”, “letter+number”, “number+space+letter”, “number+space+hour”, “number+space+h”, “month abbreviated+space+number”, “number+space+month abbreviated”, “month abbreviated.number”) ) |
| OR |
| (“vaccin” or “immuniz” or “immunis” or “booster” or “ jab” or “ shot ” or “Pfizer” or "BioNTech" or “Moderna” or “Sinovac” or “Janssen” or “Johnson & Johnson” or “AstraZeneca” or “Covishield” or “Covaxin” or “Sinovac” or “Sinopharm” or “Sputnik” |
| AND “refus” or “resistan” or “hesitan” or “inequit” or “distribution” or “inequality” or “rollout” or “maldistribution” or “side effect” or “allergic reaction” or “mandate” or “required” |
| AND professions = [     "health specialist", "health provider", "health professional", "health practitioner", "health doctor", "health worker", "health personnel", "health staff",     "healthcare specialist", "healthcare provider", "healthcare professional", "healthcare practitioner", "healthcare doctor", "healthcare worker", "healthcare personnel", "healthcare staff",     "health care specialist", "health care provider", "health care professional", "health care practitioner", "health care doctor", "health care worker", "health care personnel", "health care staff",     "medical specialist", "medical provider", "medical professional", "medical practitioner", "medical doctor", "medical worker", "medical personnel", "medical staff",     "physician", "general practitioner", "therapist", "nurse", "midwife", "caregiver", "care provider",     "gastroenterologist", "surgeon", "dentist", "psychiatrist", "pharmacist", "ophthalmologist", "cardiologist", "anaesthesiologist",     "anesthesiologist", "dermatologist", "endocrinologist", "geriatrician", "haematologist", "hematologist", "nephrologist",     "neurologist", "oncologist", "otolaryngologist", "pulmonologist", "paediatrician", "pediatrician", "radiologist", "urologist" ]) |
| 1. Exclusion |
| 3.1. Apply sentence-level contextual filtering on the body (Exclusion based on the conclusions drawn from validation process performed after each iteration of filters; keywords resulting in thematic noise): “vaccin” or “immuniz” or “immunis” or “booster” or “ jab” or “ shot ” or “Pfizer” or "BioNTech" or “Moderna” or “Sinovac” or “Janssen” or “Johnson & Johnson” or “AstraZeneca” or “Covishield” or “Covaxin” or “Sinovac” or “Sinopharm” or “Sputnik” |
| AND “patient” or “public” or “warning” or “control-study” or “training” or “preliminary data” or “administer” |
| AND professions = [     "health specialist", "health provider", "health professional", "health practitioner", "health doctor", "health worker", "health personnel", "health staff",     "healthcare specialist", "healthcare provider", "healthcare professional", "healthcare practitioner", "healthcare doctor", "healthcare worker", "healthcare personnel", "healthcare staff",     "health care specialist", "health care provider", "health care professional", "health care practitioner", "health care doctor", "health care worker", "health care personnel", "health care staff",     "medical specialist", "medical provider", "medical professional", "medical practitioner", "medical doctor", "medical worker", "medical personnel", "medical staff",     "physician", "general practitioner", "therapist", "nurse", "midwife", "caregiver", "care provider",     "gastroenterologist", "surgeon", "dentist", "psychiatrist", "pharmacist", "ophthalmologist", "cardiologist", "anaesthesiologist",     "anesthesiologist", "dermatologist", "endocrinologist", "geriatrician", "haematologist", "hematologist", "nephrologist",     "neurologist", "oncologist", "otolaryngologist", "pulmonologist", "paediatrician", "pediatrician", "radiologist", "urologist" ] |
|  |
| **Topic: Industrial action by health workforce** |
| 1. Exclusion |
| 1.1. Remove articles with sentences longer than 100 words (Articles with sentences longer than 100 are noise, e.g., news feeds) |
| 1. Inclusion: |
| 1.1. Apply inclusive filtering on the title (Ensuring that the article reports on protests/strikes rather than uses the topic as a point of reference for another topic):  “strike” or “protest” or “industrial action”  AND professions = [     "health specialist", "health provider", "health professional", "health practitioner", "health doctor", "health worker", "health personnel", "health staff",     "healthcare specialist", "healthcare provider", "healthcare professional", "healthcare practitioner", "healthcare doctor", "healthcare worker", "healthcare personnel", "healthcare staff",     "health care specialist", "health care provider", "health care professional", "health care practitioner", "health care doctor", "health care worker", "health care personnel", "health care staff",     "medical specialist", "medical provider", "medical professional", "medical practitioner", "medical doctor", "medical worker", "medical personnel", "medical staff",     "physician", "general practitioner", "therapist", "nurse", "midwife", "caregiver", "care provider",     "gastroenterologist", "surgeon", "dentist", "psychiatrist", "pharmacist", "ophthalmologist", "cardiologist", "anaesthesiologist",     "anesthesiologist", "dermatologist", "endocrinologist", "geriatrician", "haematologist", "hematologist", "nephrologist",     "neurologist", "oncologist", "otolaryngologist", "pulmonologist", "paediatrician", "pediatrician", "radiologist", "urologist" ] |
| 1. Exclusion: |
| 2.1. Apply exclusive filtering on the title (Exclusion based on the conclusions drawn from validation process performed after each iteration of filters; keywords resulting in thematic noise):  “virus strikes” or "student" or “teacher” or “lockdown” or “strike(s) vaccine” or “strike(s) COVID-19 vaccine” or “George Floyd” or “anti-vacc” or “black lives” or trump rally” |
|  |
| **Topic: Burnout, stress, and other mental health impacts on health workforce** |
| 1. Exclusion |
| 1.1. Remove articles with sentences longer than 100 words (Articles with sentences longer than 100 are noise, e.g., news feeds) |
| 2. Inclusion |
| 2.1. Apply filtering on the title (Ensuring the articles focus on HCW rather than only mention HCW in the body): professions = [     "health specialist", "health provider", "health professional", "health practitioner", "health doctor", "health worker", "health personnel", "health staff",     "healthcare specialist", "healthcare provider", "healthcare professional", "healthcare practitioner", "healthcare doctor", "healthcare worker", "healthcare personnel", "healthcare staff",     "health care specialist", "health care provider", "health care professional", "health care practitioner", "health care doctor", "health care worker", "health care personnel", "health care staff",     "medical specialist", "medical provider", "medical professional", "medical practitioner", "medical doctor", "medical worker", "medical personnel", "medical staff",     "physician", "general practitioner", "therapist", "nurse", "midwife", "caregiver", "care provider",     "gastroenterologist", "surgeon", "dentist", "psychiatrist", "pharmacist", "ophthalmologist", "cardiologist", "anaesthesiologist",     "anesthesiologist", "dermatologist", "endocrinologist", "geriatrician", "haematologist", "hematologist", "nephrologist",     "neurologist", "oncologist", "otolaryngologist", "pulmonologist", "paediatrician", "pediatrician", "radiologist", "urologist" ] |
| 2.2. Apply sentence-level contextual filtering on the body (Ensuring the article focuses on topics of interest in the context of HCW only): “burnout” or “mental health” or “mental wellness” or “anxiety” or “depression” or “PTSD” or “traumatic stress” or “suicide” or “trauma” or “fatigued” or “overburdened” or “under stress” or “exhausted” or “stressed out” or “nervous breakdown” or “depressed” or “mental health issue” or “ stress ” or “stress,” or “burned out” or “overworked” |
| AND professions = [     "health specialist", "health provider", "health professional", "health practitioner", "health doctor", "health worker", "health personnel", "health staff",     "healthcare specialist", "healthcare provider", "healthcare professional", "healthcare practitioner", "healthcare doctor", "healthcare worker", "healthcare personnel", "healthcare staff",     "health care specialist", "health care provider", "health care professional", "health care practitioner", "health care doctor", "health care worker", "health care personnel", "health care staff",     "medical specialist", "medical provider", "medical professional", "medical practitioner", "medical doctor", "medical worker", "medical personnel", "medical staff",     "physician", "general practitioner", "therapist", "nurse", "midwife", "caregiver", "care provider",     "gastroenterologist", "surgeon", "dentist", "psychiatrist", "pharmacist", "ophthalmologist", "cardiologist", "anaesthesiologist",     "anesthesiologist", "dermatologist", "endocrinologist", "geriatrician", "haematologist", "hematologist", "nephrologist",     "neurologist", "oncologist", "otolaryngologist", "pulmonologist", "paediatrician", "pediatrician", "radiologist", "urologist" ] |
| 3. Exclusion |
| 3.1.Apply sentence-level contextual filtering on the body (Exclusion based on the conclusions drawn from validation process performed after each iteration of filters; keywords resulting in thematic noise): “public anxiety” or “trauma nurse” or “trauma program” or “Surgery, Trauma” or “trauma center” or “trauma centre” or “trauma unit” or “trauma surgeon” or “trauma hospital” or “trauma service” or “trauma physician” or “in traumas” or “trauma patient” or “trauma care” or “trauma ICU nurse” or “traumatic times” or “trauma machine” or “Straumann” or “orthopedic trauma” or “trauma and orthopaedics” or “surgical trauma” “emergency trauma” or “traumatic event“ or “traumatic brain” or “trauma and emergency” or “facial trauma” or “mental health center” or “mental health centre” or “mental health team” or “mental health professional” or “governmental health” or “environmental health” or “mental health therapist” or “mental health provider” or “mental health nurse” or “mental health worker” or “mental health care provider” or “suicide mission” or “training in suicide” or “suicide nurse” or “people with” or “people deal” or “great depression” or trauma protocol” or “Mental Health” |
|  |
| **Topic: Health workforce infections and deaths** |
| 1. Exclusion |
| 1.1. Remove articles with sentences longer than 100 words (Articles with sentences longer than 100 are noise, e.g., news feeds) |
| 2. Inclusion |
| 2.1. Apply sentence-level contextual filtering on the title (Ensuring the article reports on the topic of interest rather than use it as a reference to another topic): “dead” or “death” or “died” or “infect*” or “claimed lives” or ”perished” or “passed away” or “lost live” or “decease” or “dying” or “illness” AND professions = [     "health specialist", "health provider", "health professional", "health practitioner", "health doctor", "health worker", "health personnel", "health staff",     "healthcare specialist", "healthcare provider", "healthcare professional", "healthcare practitioner", "healthcare doctor", "healthcare worker", "healthcare personnel", "healthcare staff",     "health care specialist", "health care provider", "health care professional", "health care practitioner", "health care doctor", "health care worker", "health care personnel", "health care staff",     "medical specialist", "medical provider", "medical professional", "medical practitioner", "medical doctor", "medical worker", "medical personnel", "medical staff",     "physician", "general practitioner", "therapist", "nurse", "midwife", "caregiver", "care provider",     "gastroenterologist", "surgeon", "dentist", "psychiatrist", "pharmacist", "ophthalmologist", "cardiologist", "anaesthesiologist",     "anesthesiologist", "dermatologist", "endocrinologist", "geriatrician", "haematologist", "hematologist", "nephrologist",     "neurologist", "oncologist", "otolaryngologist", "pulmonologist", "paediatrician", "pediatrician", "radiologist", "urologist" ] |
| 2.2. Apply sentence-level contextual filtering on the body (Ensuring the article reports on coverage): “dead” or “death” or “ died” or “infect*” or “contracted COVID-19” or “contracted the virus” or “tested positive” or “succumbed” or “claimed lives” or ”perished” or “passed away” or “lost live” or “decease” or “dying” or “illness” AND professions = [     "health specialist", "health provider", "health professional", "health practitioner", "health doctor", "health worker", "health personnel", "health staff",     "healthcare specialist", "healthcare provider", "healthcare professional", "healthcare practitioner", "healthcare doctor", "healthcare worker", "healthcare personnel", "healthcare staff",     "health care specialist", "health care provider", "health care professional", "health care practitioner", "health care doctor", "health care worker", "health care personnel", "health care staff",     "medical specialist", "medical provider", "medical professional", "medical practitioner", "medical doctor", "medical worker", "medical personnel", "medical staff",     "physician", "general practitioner", "therapist", "nurse", "midwife", "caregiver", "care provider",     "gastroenterologist", "surgeon", "dentist", "psychiatrist", "pharmacist", "ophthalmologist", "cardiologist", "anaesthesiologist",     "anesthesiologist", "dermatologist", "endocrinologist", "geriatrician", "haematologist", "hematologist", "nephrologist",     "neurologist", "oncologist", "otolaryngologist", "pulmonologist", "paediatrician", "pediatrician", "radiologist", "urologist" ] |
| AND one number (but ignore if “-number”, “number-“ [like in “Covid-19”, “2019-nCoV”, “SARS-CoV-2”], “number+space+min”, “number+space+sec”, “number/”, “number year old”, “number years old”, “number,+space”, “number:”, “letter+number”, “number+space+letter”, “number+space+hour”, “number+space+h”, “month abbreviated+space+number”, “number+space+month abbreviated”, “month abbreviated.number”) |
| 3. Exclusion |
| 3.1.Apply sentence-level contextual filtering on the body (Exclusion based on the conclusions drawn from validation process performed after each iteration of filters; keywords resulting in thematic noise): “deadly” or “prevent death” or “dead patient” or “infectious” AND professions = [     "health specialist", "health provider", "health professional", "health practitioner", "health doctor", "health worker", "health personnel", "health staff",     "healthcare specialist", "healthcare provider", "healthcare professional", "healthcare practitioner", "healthcare doctor", "healthcare worker", "healthcare personnel", "healthcare staff",     "health care specialist", "health care provider", "health care professional", "health care practitioner", "health care doctor", "health care worker", "health care personnel", "health care staff",     "medical specialist", "medical provider", "medical professional", "medical practitioner", "medical doctor", "medical worker", "medical personnel", "medical staff",     "physician", "general practitioner", "therapist", "nurse", "midwife", "caregiver", "care provider",     "gastroenterologist", "surgeon", "dentist", "psychiatrist", "pharmacist", "ophthalmologist", "cardiologist", "anaesthesiologist",     "anesthesiologist", "dermatologist", "endocrinologist", "geriatrician", "haematologist", "hematologist", "nephrologist",     "neurologist", "oncologist", "otolaryngologist", "pulmonologist", "paediatrician", "pediatrician", "radiologist", "urologist" ] |
| AND one number (but ignore if “-number”, “number-“ [like in “Covid-19”, “2019-nCoV”, “SARS-CoV-2”], “number+space+min”, “number+space+sec”, “number/”, “number year old”, “number years old”, “number,+space”, “number:”, “letter+number”, “number+space+letter”, “number+space+hour”, “number+space+h”, “month abbreviated+space+number”, “number+space+month abbreviated”, “month abbreviated.number”) |

**Table S2.** Health Workforce Intelligence from Open Sources (WIOS) validation rate of topic filtering on a sample of 50 news articles.

| **Topic** | **Relevance rate** |
| --- | --- |
| Infections and deaths | 88% |
| Industrial actions | 88% |
| Vaccination | 88% |
| Mental health | 72% |
| Health professionals’ education | 90% |


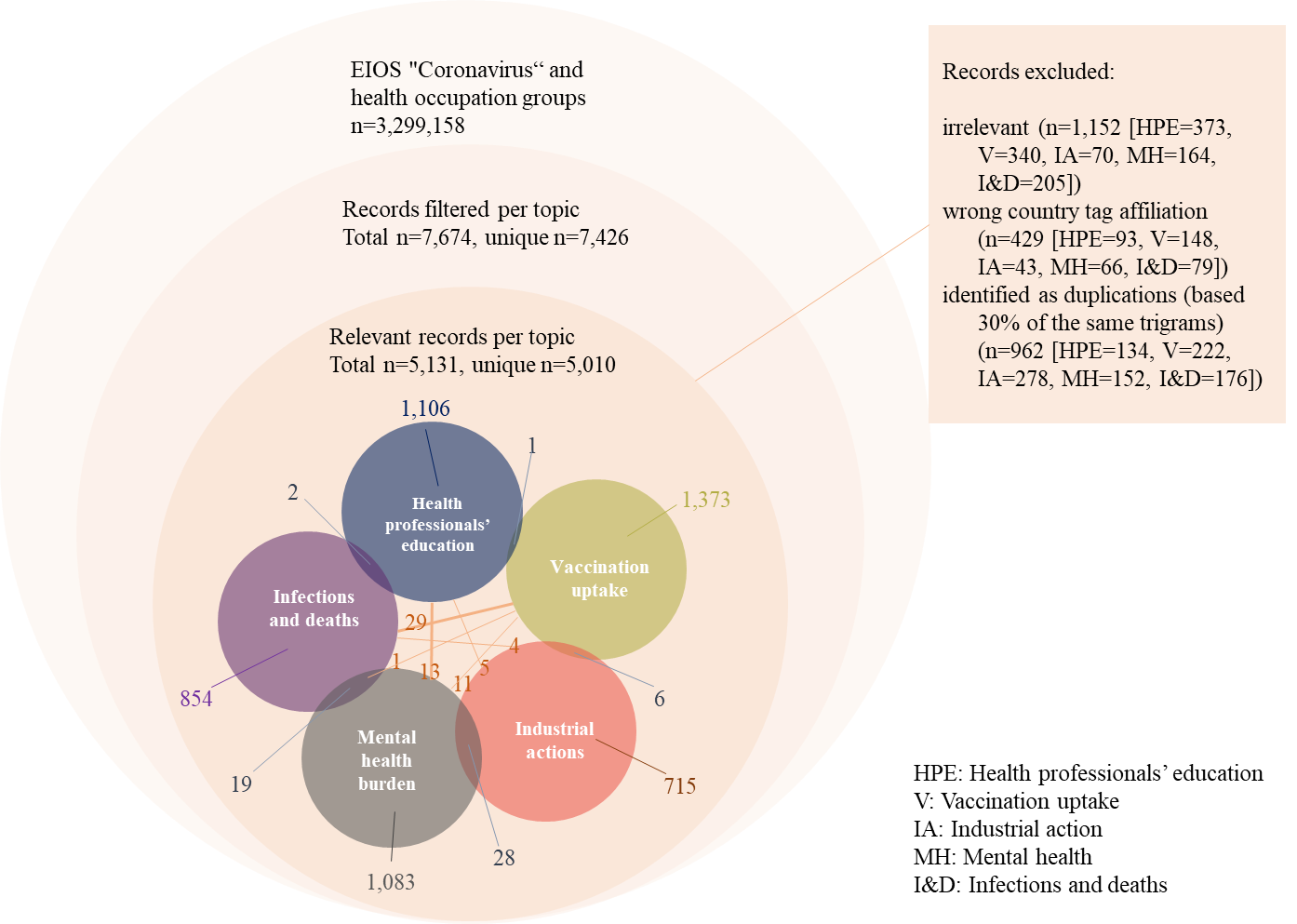


**Figure S1.** Number of news articles on COVID-19 impacts on the health workforce included in the study (January 2020-June 2022).

**Figure S2.** Number of countries with news articles on COVID-19 impacts on the health workforce according to each topic (January 2020-June 2022).


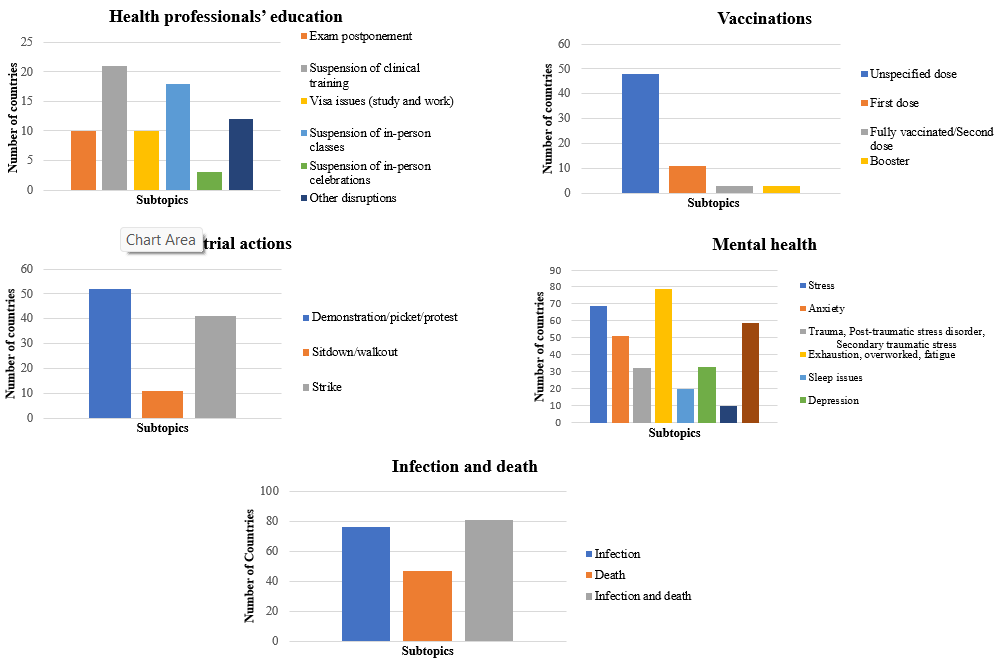


**Figure S3.** Number of countries with news articles on COVID-19 impacts on the health workforce according to sub-events of each topic (January 2020-June 2022).
